# Resting‐State EEG Functional Connectivity in Young Adults With Clinically Diagnosed Generalized Anxiety Disorder and Self‐Reported Habitual Short Sleep: An Alpha‐Band Network Analysis

**DOI:** 10.1002/brb3.71685

**Published:** 2026-08-24

**Authors:** Ce Shi, Mengyu Wang, Xinwang Chen, Jing Gao, Wen Fu, Yajing Guo, Jing Wen, Haiping Li, Run Zhang, Peidong Liu, Cong Xue, Bingzhen Li, Miaomiao Ji, Yayong He, Lihua Wu

**Affiliations:** ^1^ Henan University of Chinese Medicine Zhengzhou Henan China; ^2^ School of Rehabilitation Sciences Henan University of Chinese Medicine Zhengzhou Henan China; ^3^ College of Acupuncture and Massage Henan University of Chinese Medicine Zhengzhou Henan China; ^4^ The First Affiliated Hospital of Henan University of Chinese Medicine Zhengzhou Henan China; ^5^ The Central Hospital of Enshi Tujia and Miao Autonomous Prefecture Enshi Hubei China; ^6^ The Third Affiliated Hospital of Henan University of Chinese Medicine Zhengzhou Henan China; ^7^ Rehabilitation Campus of Luoyang Orthopedic Hospital Luoyang Henan China

**Keywords:** alpha band, EEG, functional connectivity, generalized anxiety disorder, habitual short sleep, network‐based statistics, wPLI

## Abstract

**Background:**

Generalized anxiety disorder (GAD) is commonly accompanied by sleep disturbance in young adults. Whether clinically diagnosed GAD with self‐reported habitual short sleep (HSS) is associated with altered resting‐state large‐scale functional connectivity remains unclear. We examined EEG functional connectivity in young adults with GAD + HSS and healthy controls using complementary connectivity measures and multilevel network analyses.

**Methods:**

Resting‐state EEG data were analyzed from 74 college students, including 37 participants with clinically diagnosed GAD and self‐reported HSS, defined as habitual weekday nocturnal sleep duration ≤ 6 h/night for at least 1 month, and 37 healthy controls. Functional connectivity was estimated using the weighted phase lag index (wPLI) and corrected amplitude envelope correlation (AEC‐c) across delta, theta, alpha, and beta bands. Group differences were assessed using network‐based statistics (NBS), node strength, and graph‐theoretical analyses. Covariate‐adjusted sensitivity analyses accounted for age and years of education, and graph metrics were tested across density thresholds of 10%–30%.

**Results:**

Significant group differences were confined to the alpha‐band wPLI network. NBS identified one connected component comprising 434 suprathreshold edges (FWER‐corrected p = 0.0002), predominantly showing stronger connectivity in the GAD + HSS group. All 30 scalp nodes showed higher node strength after FDR correction, including after covariate adjustment. The GAD + HSS group also showed higher global efficiency, lower characteristic path length, and higher weighted clustering coefficient, whereas transitivity did not differ. These findings were directionally stable across density thresholds.

**Conclusions:**

Young adults with clinically diagnosed GAD and self‐reported HSS showed distributed alterations in resting‐state alpha‐band phase‐based connectivity. Convergent network‐level, nodal, and graph‐theoretical findings indicated stronger weighted integration and clustering. However, these results should not be interpreted as biomarkers of objectively verified sleep loss, and the independent contributions of anxiety and sleep status cannot be determined.

## Graphical abstract

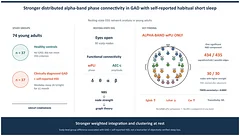

## 1 Introduction

Generalized anxiety disorder (GAD) is a common and disabling psychiatric condition in young adults, a developmental stage characterized by substantial academic, social, and emotional demands (Gilmour 2016; Tsypes et al. 2013; Yin et al. 2017). Sleep disturbance is highly prevalent in GAD, and self‐reported habitual short sleep (HSS), defined in the present study based on habitual weekday nocturnal sleep duration, may be associated with greater anxiety burden through sustained hyperarousal, impaired emotional regulation, and reduced cognitive efficiency (Bendall et al. 2024; Ferre Navarrete et al. 2017). In college populations, where habitual sleep restriction is common, the coexistence of clinically diagnosed GAD and self‐reported HSS may therefore be associated with altered resting‐state brain dynamics in young adults (Hansen et al. 2021; Witkowski et al. 2015).

Both anxiety and insufficient sleep have been associated with abnormalities in attention, vigilance, emotional processing, and executive control (Özgür et al. 2025). These alterations are unlikely to be fully explained by local neural activity alone, because GAD‐related hypervigilance, rumination, and impaired attentional disengagement, as well as sleep‐loss‐related disturbances in vigilance, arousal regulation, and cognitive control, are inherently network‐level phenomena (Hao et al. 2024; C. Li et al. 2019; van Dellen et al. 2013). Electroencephalography (EEG), with its high temporal resolution, offers a useful approach for probing such abnormalities (Fellinger et al. 2011; Stokes and Prerau 2020). Although previous EEG studies in anxiety‐ and sleep‐related conditions have focused mainly on spectral power, power‐based analyses primarily reflect the magnitude of local oscillatory activity and do not directly characterize interregional functional coordination (Y. Li et al. 2016; Perera et al. 2023).

Resting‐state functional connectivity analysis may therefore provide additional insight into the neural organization of clinically diagnosed GAD accompanied by self‐reported HSS. The weighted phase lag index (wPLI) quantifies phase‐based synchronization while reducing the influence of volume conduction, whereas corrected amplitude envelope correlation (AEC‐c) measures amplitude‐based coupling (Candelaria‐Cook and Stephen 2020; Chen et al. 2025). Because these measures capture different aspects of neural communication, their combined use may clarify whether connectivity differences in GAD + HSS are generalized or more specific to particular bands and coupling modes. Alpha‐band connectivity is of particular interest because alpha oscillations are closely related to cortical inhibition, internally directed attention, vigilance regulation, and large‐scale coordination of resting‐state processing (Cao et al. 2020; Magosso et al. 2021; Pellegrino et al. 2021).

Accordingly, the present study examined resting‐state EEG functional connectivity in young adults with clinically diagnosed GAD and self‐reported HSS based on habitual weekday nocturnal sleep duration, compared with healthy controls (HCs), using wPLI and AEC‐c in the delta, theta, alpha, and beta bands. Network‐based statistics (NBS) were used to identify network‐level group differences, node strength analysis was performed to assess the spatial distribution of altered connectivity, and graph‐theoretical analysis was applied to further characterize the topology of the principal abnormal network. We hypothesized that the GAD + HSS group would show altered resting‐state functional connectivity relative to HCs, with the most prominent differences expected in alpha‐band phase‐based synchronization.

## 2 Methods

### 2.1 Participants

This study included 74 young adults, comprising 37 participants with clinically diagnosed GAD and self‐reported HSS, defined by habitual weekday nocturnal sleep duration (GAD + HSS group) and 37 HCs. Participants were recruited from the Longzihu Campus and Dongming Road Campus of Henan University of Chinese Medicine between June 2023 and December 2024. The diagnosis of GAD was established according to the International Statistical Classification of Diseases and Related Health Problems, 10th Revision (ICD‐10), through a clinical assessment conducted by a licensed clinical psychologist. The clinical psychologist had received systematic training in the application of the ICD‐10 criteria for GAD, and the assessment consisted of a face‐to‐face clinical interview combined with questionnaire‐based evaluation. The GAD‐7 was not used as the sole diagnostic basis; rather, a score of ≥ 5 was used as an additional symptom‐severity criterion to confirm the presence of current anxiety symptoms among participants who had already met ICD‐10 diagnostic criteria for GAD. In the present study, HSS was defined as self‐reported habitual weekday nocturnal sleep duration of ≤ 6 h/night persisting for at least 1 month. The ≤ 6 h/night threshold represents sustained weekday nocturnal sleep duration below the ≥ 7 h per night generally recommended for healthy adults by the American Academy of Sleep Medicine and Sleep Research Society (Watson et al. 2015). This threshold was selected as a population‐based criterion for self‐reported HSS and does not imply that all individuals reporting ≤ 6 h/night necessarily had a sleep disorder or objectively verified sleep loss. The duration criterion of at least 1 month was a prespecified research criterion. The GAD + HSS classification was used for research grouping and was not intended to constitute a formal clinical diagnosis of a sleep disorder or objectively verified sleep loss. Sleep duration was assessed retrospectively by self‐report, without a prospective sleep diary, actigraphy, or polysomnography.

The inclusion criteria for the GAD + HSS group were as follows: age 18–25 years; fulfillment of the ICD‐10 diagnostic criteria for GAD based on clinical assessment by a clinical psychologist; a Generalized Anxiety Disorder 7‐Item Scale (GAD‐7) score ≥ 5 as an additional symptom‐severity criterion; self‐reported habitual weekday nocturnal sleep duration of ≤ 6 h/night persisting for at least 1 month; no anti‐anxiety medication use or anti‐anxiety acupuncture treatment within the previous 30 days; and provision of written informed consent. Exclusion criteria included organic mental disorders, anxiety, or depression secondary to surgery or psychoactive substances, a family history of severe psychiatric disorders such as dementia, schizophrenia, mania, or hysteria, and cognitive impairment that would interfere with EEG acquisition. HCs were recruited from the same source population and underwent a corresponding face‐to‐face clinical interview and questionnaire‐based assessment. They were required to be 18–25 years old, not to meet the ICD‐10 diagnostic criteria for GAD or other major psychiatric disorders, not to meet the study's HSS criterion based on self‐reported habitual weekday nocturnal sleep duration, and to be able to complete EEG acquisition; they were also required to provide written informed consent. As in the GAD + HSS group, sleep status in HCs was based on self‐report and was not objectively verified.

This study was approved by the Ethics Committee of the Third Affiliated Hospital of Henan University of Chinese Medicine (approval no. 2023SH‐147). All participants provided written informed consent before enrollment, and all procedures were conducted in accordance with the Declaration of Helsinki.

### 2.2 EEG Acquisition

Resting‐state EEG data were acquired using a 32‐channel actiCHamp plus EEG system (Brain Products, Germany) with Ag/AgCl scalp electrodes. Online filtering was set at 0.1–100 Hz, and electrode impedance was maintained at ≤ 5 kΩ before recording. EEG acquisition was performed in a quiet, dimly lit room under controlled environmental conditions. Participants were instructed to avoid alcohol, caffeine‐containing foods or beverages, and moderate‐to‐vigorous exercise during the 24 h before testing. On the day of recording, participants entered the laboratory 30 min in advance to adapt to the environment. During EEG acquisition, they were instructed to remain seated, keep their eyes open, gaze at the computer monitor, and minimize body movement.

### 2.3 EEG Preprocessing and Current Source Density Transformation

EEG preprocessing was performed in EEGLAB. Raw EEG data in Brain Products format (.vhdr) were first imported into EEGLAB, and scalp electrode locations were assigned using a standard 10–5 electrode template. The continuous EEG data were band‐pass filtered at 0.5–30 Hz and notch‐filtered at 48–52 Hz to suppress line‐noise contamination. The data were then downsampled to 250 Hz (Janardhan Reddy and Ramasubba Reddy 2024; Leske and Dalal 2019).

A multistep re‐referencing procedure was applied during preprocessing. After intermediate reference operations used for channel reconstruction and montage standardization, the EEG data were ultimately re‐referenced to the bilateral mastoid electrodes (TP9 and TP10). Channels not included in the final montage were removed, and missing channels required for the final electrode configuration were appended and localized before re‐referencing. The continuous resting‐state EEG data were then segmented into consecutive 2‐s epochs without baseline correction.

After segmentation, the epoched EEG data were visually inspected for non‐stereotyped artifacts and poor‐quality channels. Epochs containing obvious gross artifacts, including large‐amplitude movement‐related interference, prominent drift, unstable high‐frequency noise, or other non‐physiological signal contamination, were manually rejected. Channels were considered bad when they showed persistently abnormal waveforms across epochs, such as sustained excessive noise, flat or near‐flat activity, intermittent signal loss, or marked deviation from neighboring electrodes. Bad channels identified during this stage were interpolated using information from surrounding electrodes before subsequent ICA decomposition. All enrolled participants retained sufficient valid 2‐s epochs for the predefined preprocessing and connectivity pipeline, and no participant in either group was excluded for insufficient valid data.

Independent component analysis (ICA) was then performed using the extended Infomax algorithm. Independent components were reviewed manually with the assistance of ICLabel. Components were removed only when they were judged to represent non‐neural artifacts on the basis of their spatial distribution, spectral profile, and temporal characteristics. On average, 7.6 ± 1.8 independent components were removed in the GAD + HSS group and 7.3 ± 1.6 in the HC group. Components judged to reflect plausible brain activity were retained. The cleaned data were then reconstructed for subsequent analysis.

Although EEG data were acquired using a 32‐channel montage, the final functional connectivity analyses were conducted on 30 scalp nodes. This is because the bilateral mastoid electrodes (TP9 and TP10) were used as the final reference electrodes and were therefore excluded from subsequent network construction and graph‐based analyses.

To improve spatial specificity and reduce the influence of volume conduction in connectivity estimation, a current source density (CSD) transformation was applied to the final preprocessed epoched EEG datasets before functional connectivity analysis (Tsuchimoto et al. 2021). CSD transformation was performed in batch mode using a standard scalp montage file (10‐5‐System_Mastoids_EGI129.csd). The first valid dataset was used to define a template channel list, and all subsequent datasets were checked, reordered, and restricted to this common channel template before CSD computation. Channel labels were harmonized across datasets using standard legacy‐to‐modern conversions when needed (e.g., T3/T4/T5/T6 to T7/T8/P7/P8; FCZ/CPZ/OZ to FCz/CPz/Oz).

The spherical spline transformation matrices were computed once from the montage and then applied to each dataset. For epoched EEG data, the CSD transform was applied separately to each epoch. Because the CSD transform changes the data space, previously estimated ICA weights were considered invalid and were removed from the transformed datasets. The resulting CSD‐transformed datasets were then used for functional connectivity analysis.

### 2.4 Functional Connectivity Estimation

Functional connectivity was estimated separately for the delta (1–4 Hz), theta (4–7 Hz), alpha (8–13 Hz), and beta (13–30 Hz) bands using two complementary measures: the wPLI and corrected AEC‐c. Connectivity was computed from CSD‐transformed epoched EEG data.

For each frequency band, band‐pass filtering was performed using EEGLAB‐compatible finite impulse response filtering, preferentially with pop_eegfiltnew and with automatic adjustment of filter order when required by the data length (Mori et al. 2012). When this function was unavailable, legacy eegfilt was used as a fallback. After filtering, the analytic signal was obtained using the Hilbert transform. To reduce filter‐ and Hilbert‐related edge effects, 0.5 s was trimmed from both ends of each epoch before connectivity estimation (Marzetti et al. 2024).

For wPLI, phase‐based synchronization was calculated edge‐wise using the imaginary component of the cross‐spectrum derived from the analytic signals, pooled across all retained time points and epochs. For each channel pair, wPLI was computed as the absolute value of the summed imaginary cross‐product divided by the sum of the absolute imaginary cross‐products across all retained samples (Thuraisingham 2023). This procedure yielded a symmetric weighted connectivity matrix for each participant within each frequency band.

AEC‐c was computed as a symmetric orthogonalized envelope correlation. First, the analytic amplitude envelope was extracted from each channel, and a logarithmic transform of the envelope was applied before correlation analysis. For each channel pair within each epoch, orthogonalization was performed in both directions (Hipp et al. 2012). The envelope of the orthogonalized real‐valued signal was then reconstructed using the Hilbert transform, and Pearson correlations were calculated between the original log‐envelope of one channel and the orthogonalized log‐envelope of the other channel (Nolte et al. 2020). The two directional correlation coefficients were averaged to obtain a symmetric AEC‐c estimate. Epoch‐wise AEC‐c matrices were then averaged across epochs for each participant.

For each participant, each measure, and each frequency band, a symmetric channel × channel connectivity matrix was obtained, with diagonal entries set to zero. These individual matrices were subsequently stacked into channel × channel × subject arrays for group‐level statistical analysis.

### 2.5 Network‐Based Statistics

Group differences in functional connectivity were assessed using NBS separately for wPLI and AEC‐c in each frequency band (Zalesky et al. 2010). The comparison was performed between HCs and the GAD + HSS group using an independent‐samples design. Before statistical analysis, all connectivity matrices were cleaned by handling non‐finite values, symmetrizing each matrix, and setting the diagonal to zero. NBS was used as the primary unadjusted network‐level inferential analysis.

For each channel pair, a pooled‐variance independent‐samples *t* statistic was calculated. A primary threshold corresponding to a two‐sided uncorrected *p* value of 0.05 was converted to the equivalent absolute *t* threshold based on the degrees of freedom of the between‐group comparison. Suprathreshold edges were assembled into connected components, and component size was defined using extent, that is, the number of edges within each connected component. Statistical significance of each observed component was evaluated using 5000 label permutations. For each permutation, group labels were randomly shuffled, edge‐wise *t* statistics were recalculated, the largest connected component under the same threshold was identified, and its extent was retained as the maximal null statistic. Family‐wise error rate (FWER)‐corrected *p* values for observed components were calculated as the proportion of permutations in which the maximal null component extent was greater than or equal to the observed component extent.

### 2.6 Node Strength Analysis

Because the NBS results indicated that the principal abnormality was confined to the alpha‐band wPLI network, subsequent nodal analysis was restricted to alpha‐band wPLI matrices. For each participant, node strength was defined as the sum of all edge weights connected to a given node after symmetrization of the connectivity matrix and removal of diagonal elements. This yielded one nodal strength value per channel for each participant.

Between‐group comparisons of nodal strength were first performed at each scalp node. Because age and years of education differed between groups at baseline, covariate‐adjusted sensitivity analyses were additionally performed using linear regression models with node strength as the dependent variable, group as the predictor of interest, and age and years of education as covariates. Two‐sided *p* values for the group effect were computed, and multiple comparisons across nodes were controlled using the Benjamini–Hochberg false discovery rate (FDR) procedure with *q* < 0.05. Topographic maps of channel‐wise *t* values were generated to visualize the spatial distribution of nodal abnormalities.

### 2.7 Graph‐Theoretical Analysis

Because the network‐level and nodal findings converged on alpha‐band wPLI abnormalities, graph‐theoretical analysis was also restricted to the alpha‐band wPLI matrices. For each participant, the connectivity matrix was cleaned by removing non‐finite values, symmetrizing the matrix, setting the diagonal to zero, and replacing negative values with zero. To ensure comparability across participants, a fixed‐density thresholding approach was applied. The primary analysis retained the strongest 20% of upper‐triangular edges and reconstructed a symmetric thresholded network. To assess robustness to threshold selection, the same analysis was repeated across a range of densities (10%, 15%, 20%, 25%, and 30%).

Four graph‐theoretical metrics were calculated for each participant: weighted global efficiency (*E*_glob_), weighted characteristic path length (*L*_char_), weighted clustering coefficient based on the Onnela formulation (*C*_w_), and binary transitivity. Weighted shortest‐path distances were defined as the inverse of edge weights. Global efficiency was computed as the mean inverse shortest‐path distance across all node pairs, whereas characteristic path length was defined as the mean finite shortest‐path distance. Weighted clustering coefficient quantified local clustering in the weighted network, and transitivity was computed on the binarized thresholded graph as a global measure of triangle closure.

Between‐group differences in graph metrics were first assessed at the primary 20% density threshold. Because age and years of education differed between groups, covariate‐adjusted sensitivity analyses were then performed for each graph metric using linear regression models with group as the predictor of interest and age and years of education as covariates. *p* values across the four graph metrics were corrected using the Benjamini–Hochberg FDR procedure, with *q* < 0.05 considered statistically significant. The same covariate‐adjusted analysis was repeated across all tested density thresholds to evaluate threshold robustness.

### 2.8 Statistical Analysis

Demographic and clinical variables were compared between groups using independent‐samples *t* tests for continuous variables and the chi‐square test for sex distribution. For connectivity analyses, statistical significance was evaluated at the network level using permutation‐based FWER correction for NBS and at the nodal and graph‐metric levels using FDR correction. Covariate‐adjusted sensitivity analyses were performed for node strength and graph‐theoretical metrics using linear regression models with age and years of education as covariates. All tests were two‐sided, and *p* < 0.05 was considered statistically significant unless otherwise specified.

## 3 Results

### 3.1 Participant Characteristics

A total of 74 young adults were included in the final analysis, comprising 37 participants with clinically diagnosed GAD and self‐reported HSS based on habitual weekday nocturnal sleep duration and 37 HCs. The GAD + HSS group was younger than the HC group (20.16 ± 2.60 vs. 21.30 ± 0.88 years, *p* = 0.014) and had fewer years of education (14.03 ± 2.27 vs. 15.24 ± 0.83 years, *p* = 0.003). The GAD + HSS group also had higher GAD‐7, HAMA, and SDI values than the HC group (all *p* < 0.001). Sex distribution did not differ significantly between groups (*p* = 0.140). Participant characteristics are summarized in Table 1.

**TABLE 1:** Baseline characteristics of participants.

Characteristics	GAD + HSS (*n* = 37)	HC (*n* = 37)	*t*/*χ* ^2^	*p* value
Age (years)	20.16 ± 2.60	21.30 ± 0.88	−2.52	0.014
Gender, male/female	9/28	16/21	2.17	0.140
Education (years)	14.03 ± 2.27	15.24 ± 0.83	−3.06	0.003
GAD‐7	11.05 ± 3.15	2.54 ± 2.39	13.09	< 0.001
HAMA	21.03 ± 7.60	3.92 ± 3.04	12.71	< 0.001
SDI	1.95 ± 0.67	1.25 ± 0.18	6.15	< 0.001

*Note*: Data are presented as mean ± SD unless otherwise indicated. SDI calculated as the difference between mean hours of sleep on weekends and mean hours of sleep on weekdays. The SDI was a derived descriptive variable based on self‐reported sleep duration and was not a validated standalone sleep questionnaire, prospective sleep diary, or objective confirmation of habitual short sleep. Sex was compared using the chi‐square test; continuous variables were compared using independent‐samples *t* tests.

Abbreviation: SDI = Sleep Duration Index.

### 3.2 NBS Results

Across all band‐ and measure‐specific NBS analyses, a significant between‐group difference was identified only in the alpha‐band wPLI network. No significant connected components were observed in the delta, theta, or beta wPLI networks, and no significant components were detected in any AEC‐c network.

**FIGURE 1:**
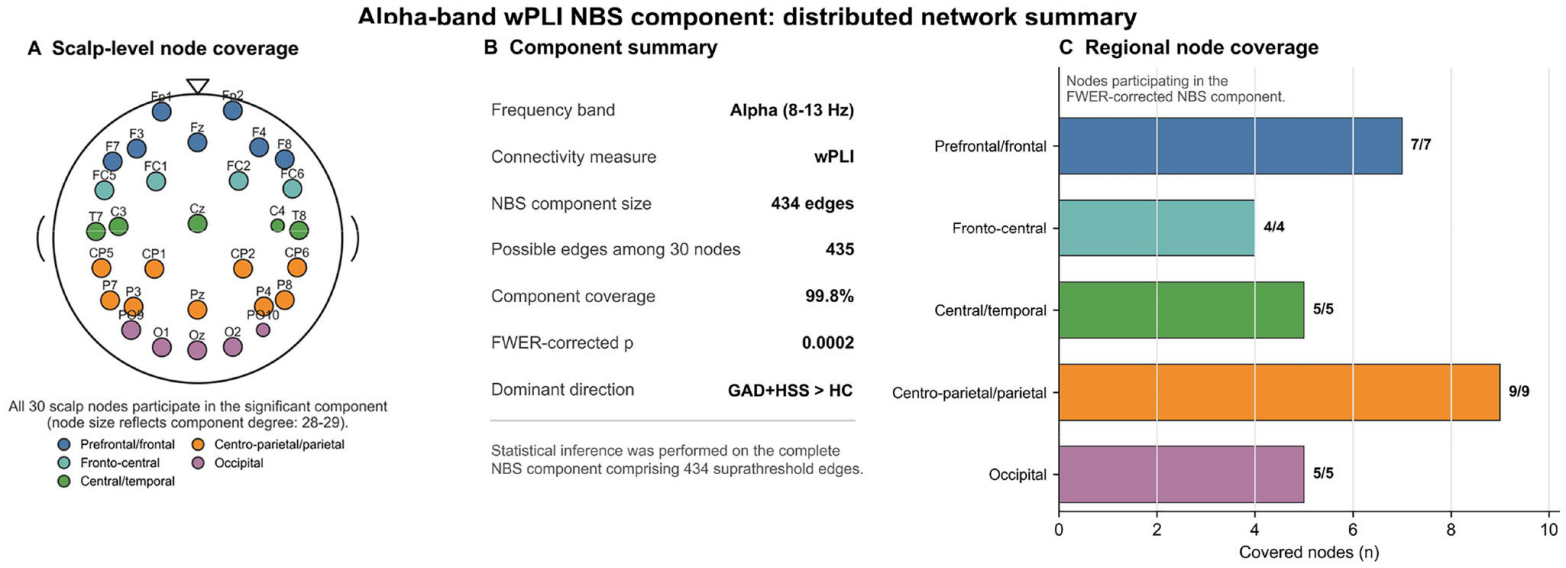
Widespread alpha‐band wPLI network difference identified by network‐based statistics.

For alpha‐band wPLI, NBS revealed one significant connected component with a FWER‐corrected *p* value of 0.0002, comprising 434 suprathreshold edges. The dominant direction of effect indicated stronger connectivity in the GAD + HSS group than in HCs. The spatial distribution and summary characteristics of the significant alpha‐band wPLI component are shown in Figure 1. The complete edge‐level display of the 434‐edge component is provided as Figure S1.

NBS identified one significant connected component in the alpha‐band wPLI network when comparing HCs and participants with clinically diagnosed GAD and self‐reported HSS based on habitual weekday nocturnal sleep duration (GAD + HSS). The component survived FWER correction and comprised 434 suprathreshold edges. To improve visual interpretability, the revised figure summarizes the spatial organization of the component using scalp‐level node coverage, component‐size information, and regional node coverage. The complete edge‐level display of the full NBS component is provided as Figure S1. All statistical inference was performed on the complete NBS component; the simplified main figure was used only for visualization. The component was predominantly characterized by stronger alpha‐band phase synchronization in the GAD + HSS group than in HC and involved distributed prefrontal/frontal, fronto‐central, central/temporal, centro‐parietal/parietal, and occipital scalp regions.

### 3.3 Node Strength Results

**FIGURE 2:**
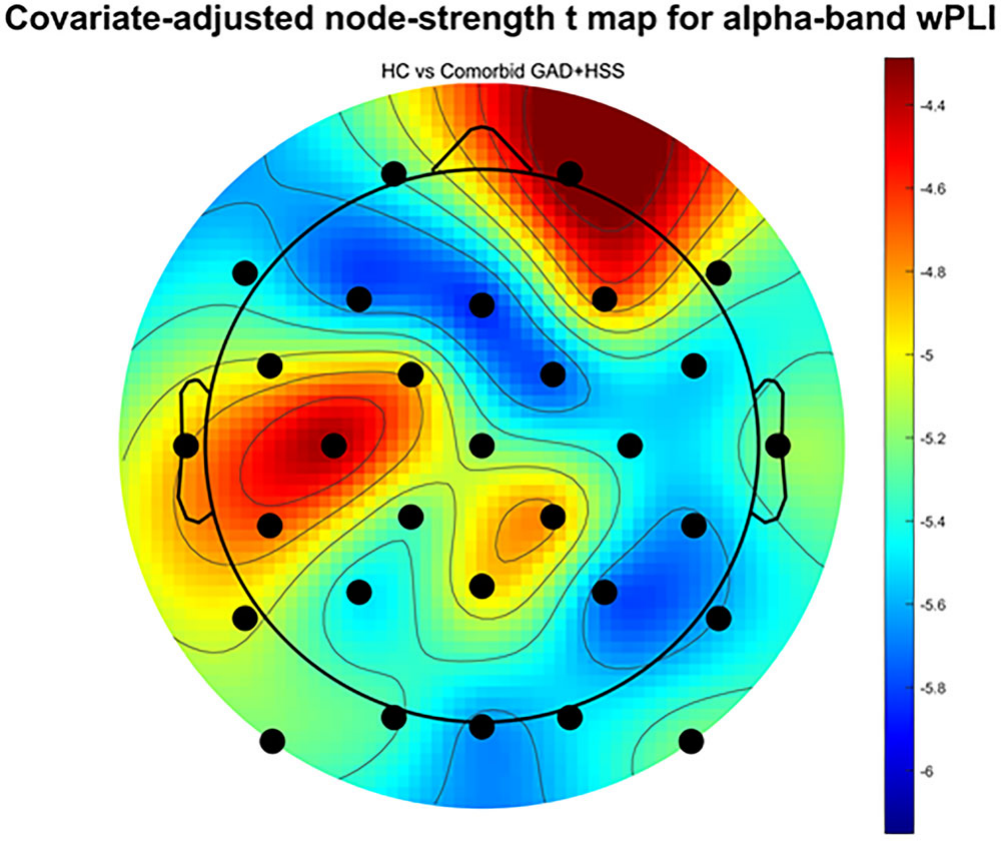
Covariate‐adjusted node‐strength *t* map for alpha‐band wPLI.

Because the network‐level abnormality was restricted to alpha‐band wPLI, subsequent node strength analysis was limited to alpha‐band wPLI matrices. The results showed a spatially widespread rather than focal effect, with all 30 analyzed nodes surviving FDR correction in the primary analysis and remaining significant after adjustment for age and years of education. The spatial distribution of covariate‐adjusted node‐strength differences is shown in Figure 2.

Topographic map of channel‐wise *t* values from covariate‐adjusted linear regression models examining node strength in the alpha‐band wPLI network. Models included group as the predictor of interest and age and years of education as covariates. Node strength was defined as the sum of all edge weights connected to each channel after matrix symmetrization and removal of diagonal elements. Multiple comparisons across scalp nodes were controlled using the Benjamini–Hochberg FDR procedure. The spatial pattern shows broadly distributed nodal differences, with higher node strength in the GAD + HSS group than in HCs across most scalp regions.

Negative values in the topographic map reflected the direction of the contrast (HCs − GAD + HSS) and therefore corresponded to broadly higher node strength in the GAD + HSS group.

### 3.4 Graph‐Theoretical Analysis

**FIGURE 3:**
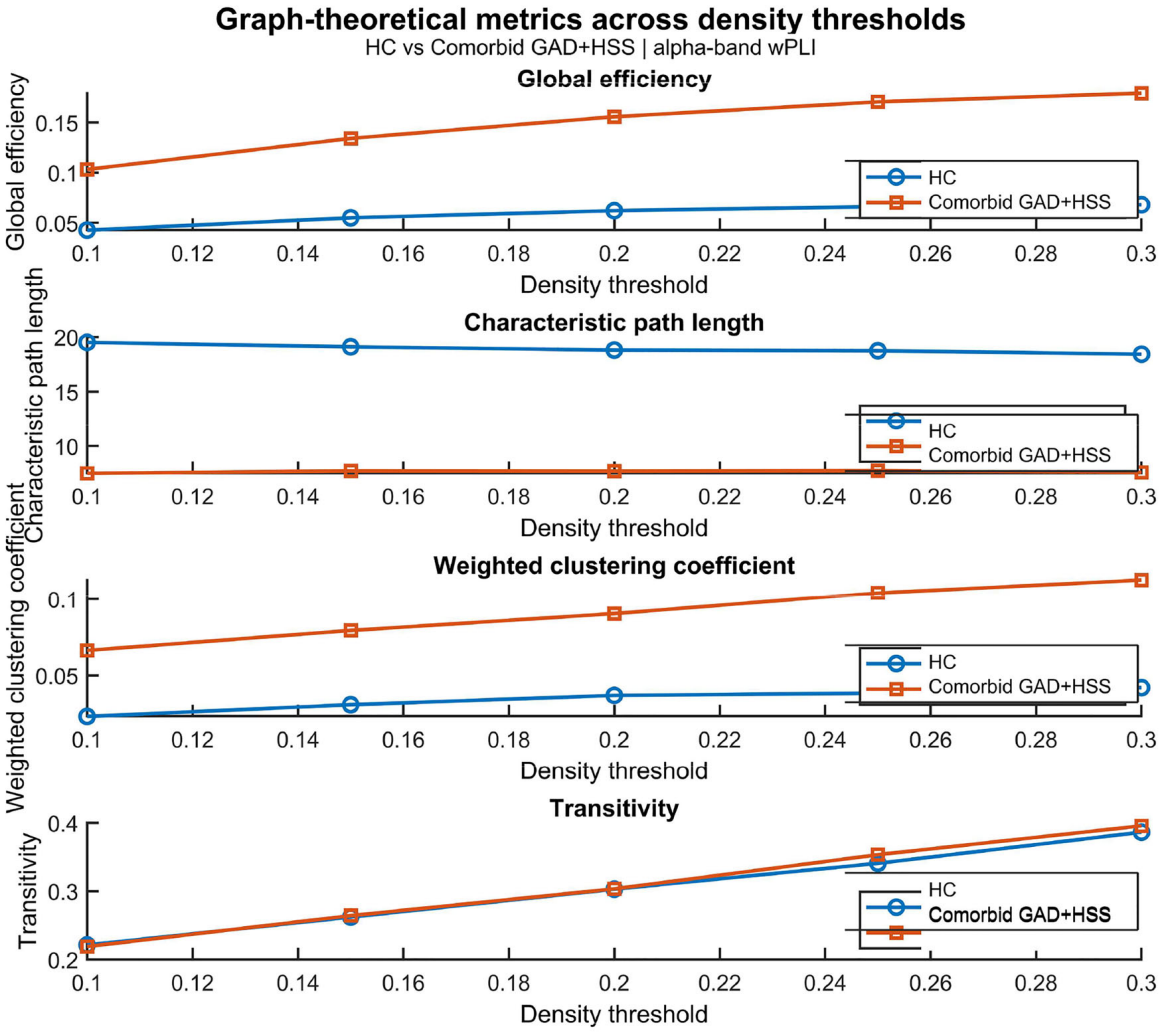
Graph‐theoretical metrics across density thresholds in the alpha‐band wPLI network.

Because both the NBS and node strength analyses converged on alpha‐band wPLI abnormalities, graph‐theoretical analysis was also restricted to the alpha‐band wPLI network. At the primary 20% density threshold, the GAD + HSS group showed higher global efficiency, lower characteristic path length, and higher weighted clustering coefficient than HCs, whereas transitivity showed no significant group difference. The same directional pattern remained after adjustment for age and years of education and was stable across density thresholds from 10% to 30%. The graph‐theoretical results across density thresholds are shown in Figure 3.

Graph‐theoretical analysis was restricted to the alpha‐band wPLI network because both the network‐level and nodal analyses converged on abnormalities in this measure and frequency band. The primary between‐group comparison was performed at the 20% density threshold, and the 10%–30% range was used to assess threshold robustness. Mean values are shown for HCs and participants in the GAD + HSS group (clinically diagnosed GAD with self‐reported HSS based on habitual weekday nocturnal sleep duration) across proportional density thresholds of 10%, 15%, 20%, 25%, and 30%. The four displayed metrics were weighted global efficiency (*E*_glob_), weighted characteristic path length (*L*_char_), weighted clustering coefficient based on the Onnela formulation (*C*_w_), and binary transitivity. Across tested densities, the GAD + HSS group consistently showed higher *E*_glob_ and *C*_w_ together with lower *L*_char_ than HC, whereas transitivity showed no clear between‐group difference.

## 4 Discussion

### 4.1 Principal Findings

Across all frequency bands and connectivity measures examined, the only robust between‐group difference emerged in alpha‐band phase‐based synchronization as indexed by wPLI. This group difference was not limited to a single edge or scalp region. NBS identified a 434‐edge connected component that survived FWER correction, node strength differed at all 30 analyzed scalp nodes, and graph‐theoretical metrics showed a consistent alteration in alpha‐band network organization. The absence of corresponding findings in AEC‐c or in other frequency bands argues against a nonspecific increase in functional coupling. Instead, the results suggest that the combined GAD + HSS research classification is preferentially associated with a distributed alteration in alpha‐band phase coordination.

This finding is clinically relevant because the main symptoms of GAD and persistent sleep restriction are not easily explained by isolated local cortical changes. Persistent worry, hypervigilance, inefficient disengagement from internal thoughts, and unstable vigilance regulation all depend on distributed brain‐state organization. The present results therefore provide a network‐level account of GAD + HSS, in which alpha‐band phase coupling is more strongly expressed across the scalp‐level resting‐state network.

### 4.2 Alpha‐Band Phase Synchronization and Symptom Relevance

The selective involvement of alpha‐band wPLI has specific implications. Alpha oscillations are closely related to cortical inhibition, vigilance regulation, and the allocation of internally versus externally directed attention. Alpha‐band phase synchrony has also been linked to large‐scale control networks and adaptive coordination of distributed cortical regions during rest and cognition (Magosso et al. 2021; Sadaghiani et al. 2019, 2012). Within this framework, stronger alpha‐band wPLI connectivity in the GAD + HSS group may indicate a resting‐state configuration in which distributed regions remain more tightly phase‐coupled than in HCs, even in the absence of explicit task demands.

This pattern is compatible with several clinical features of GAD. Patients with GAD often have difficulty releasing attention from threat‐related or ruminative content, and this difficulty may persist even when external demands require a shift of attention. Stronger alpha‐band phase coupling across distributed scalp regions may therefore reflect a neural condition that favors persistent internal mentation and heightened monitoring. It should not be taken as evidence that alpha synchronization directly causes worry; rather, it suggests that the resting brain in GAD + HSS may be organized in a way that supports attentional rigidity.

The sleep‐related component of the GAD + HSS classification may also be associated with this pattern. Self‐reported HSS meeting the HSS criterion can be associated with disturbances in arousal regulation, cognitive efficiency, and vigilance stability during wakefulness. Under such conditions, broader phase coordination may reflect an altered arousal state. When clinically diagnosed GAD and self‐reported HSS coexist, anxiety‐related hypervigilance, and sleep‐related arousal dysregulation may reinforce one another, producing a more pronounced alpha‐band synchronization phenotype. Because the present design was cross‐sectional and included only a combined patient group, this interpretation remains hypothetical and cannot be separated into independent anxiety‐related and sleep‐related contributions.

A further caution is needed. The present study did not directly test associations between symptom severity and connectivity or graph‐theoretical metrics. The proposed symptom‐level interpretation should therefore be regarded as a network‐level interpretation of the group difference, not as direct evidence of symptom‐connectivity coupling.

### 4.3 Interpretation of Graph‐Theoretical Findings

Graph‐theoretical analysis helps translate the edge‐level connectivity findings into properties of network organization. Higher global efficiency and lower characteristic path length indicate a more integrated alpha‐band wPLI network in the GAD + HSS group. In a resting‐state clinical context, however, stronger integration should not be interpreted simply as better neural efficiency. A network that is highly integrated during undirected rest may have less room to reconfigure when cognitive demands change. The present pattern may therefore reflect an over‐integrated resting configuration rather than an adaptive improvement in information processing.

The higher weighted clustering coefficient adds an important local dimension. It suggests that neighboring or functionally related nodes were also more strongly coupled within local clusters. The coexistence of higher global efficiency and higher weighted clustering indicates that the GAD + HSS group showed stronger weighted coordination at both global and local levels. Such a configuration is consistent with a less flexible network state that may sustain repetitive internally oriented processing, heightened monitoring, and inefficient attentional disengagement.

The lack of a significant difference in binary transitivity helps constrain this interpretation. Transitivity was calculated on the binarized thresholded graph, whereas global efficiency, characteristic path length, and weighted clustering coefficient were sensitive to connection weights. The present pattern therefore suggests that the abnormality was expressed mainly in the strength of existing functional connections, not in the emergence of a fundamentally different binary network backbone. In this sense, the GAD + HSS group appears to show a weighted coordination shift within the alpha‐band network rather than a wholesale reorganization of network architecture.

The directional stability of the graph‐theoretical findings across density thresholds from 10% to 30% further supports the interpretation. Findings driven by a narrow threshold choice often weaken or reverse when the density is varied. Here, the same direction of group differences was maintained across a moderate range of thresholds, suggesting that the altered topology reflects a general feature of the alpha‐band wPLI network rather than a property of a small subset of edges.

### 4.4 Interpretation of the Widespread Alpha‐Band Component

The size and spatial extent of the NBS component require careful interpretation. The component comprised 434 suprathreshold edges, which is close to the 435 possible undirected connections among 30 scalp nodes. This does not mean that every cortical region was anatomically involved, nor does it provide precise localization of source‐level networks. It indicates that the group difference in alpha‐band wPLI was broadly distributed across the scalp‐level connectivity matrix.

There are plausible neurophysiological reasons for such a broad pattern. Alpha oscillations are not restricted to a single cortical area. They participate in large‐scale resting‐state coordination, inhibitory regulation, vigilance control, and the balance between internal and external attention. A shift in alpha‐band phase synchronization can therefore appear as a distributed network‐level effect rather than as a focal abnormality. This is particularly relevant here because clinically diagnosed GAD and self‐reported HSS meeting the HSS criterion both involve disturbances in global brain‐state regulation. GAD is associated with persistent worry, threat monitoring, and impaired disengagement from internal thoughts, whereas the sleep‐related component of the GAD + HSS classification may be associated with altered arousal regulation and reduced cognitive efficiency. Their coexistence may therefore produce a broad resting‐state connectivity signature.

The statistical nature of NBS also matters. NBS identifies connected components formed by suprathreshold edges; it does not require a single focal source of abnormality. A large component can emerge when many distributed connections show moderate group differences in the same direction. In the present study, this interpretation is supported by the nodal and graph‐level findings: node strength differed across all analyzed scalp nodes, and graph‐theoretical analysis showed altered weighted integration and clustering. These convergent results support a distributed alpha‐band phase‐synchronization abnormality, while also cautioning against overinterpreting the component as anatomically specific.

### 4.5 Measure Specificity: WPLI‐Positive and AEC‐c‐Negative Findings

The absence of significant AEC‐c findings helps define the nature of the observed abnormality. wPLI and AEC‐c capture different aspects of functional coupling. wPLI reflects the consistency of non‐zero‐lag phase relationships and is relatively resistant to spurious zero‐lag coupling, whereas AEC‐c reflects amplitude‐envelope cofluctuation after orthogonalization. The present dissociation suggests that the main group difference was expressed preferentially in the timing of alpha‐band oscillatory cycles rather than in shared amplitude fluctuations.

This specificity reduces the likelihood that the result reflects a broad, nonspecific increase in all forms of EEG connectivity. If both wPLI and AEC‐c had shown widespread increases, the interpretation would be less precise and more vulnerable to nonspecific explanations. Instead, the current pattern points to alpha‐band phase coordination as a sensitive feature of resting‐state network alteration in GAD + HSS. This conclusion should remain measure‐specific and should not be generalized to all forms of neural communication.

### 4.6 Limitations

Several limitations should be acknowledged. First, although the sample size was adequate to detect consistent group differences in the present analyses, it was still modest for a resting‐state EEG network study. The stability of the alpha‐band connectivity pattern therefore needs to be confirmed in larger independent samples.

A second limitation concerns the characterization of anxiety severity. GAD diagnosis was established according to ICD‐10 criteria through clinical assessment by a clinical psychologist, and the GAD‐7 was not used as the sole diagnostic basis. However, the GAD‐7 threshold used for inclusion was relatively low and allowed participants with mild current anxiety symptom severity to be included. Future studies with larger samples could further examine whether the present EEG findings vary across mild, moderate, and severe anxiety symptom levels.

Sleep status should be interpreted cautiously. In this study, HSS was a self‐report‐based research classification defined as habitual weekday nocturnal sleep duration of ≤ 6 h/night persisting for at least 1 month. Although this threshold represents sustained sleep duration below the amount generally recommended for healthy adults by the American Academy of Sleep Medicine and Sleep Research Society (Watson et al. 2015), that population‐level recommendation is not a formal sleep‐disorder diagnostic standard. Individual habitual baseline sleep and physiological sleep need for optimal performance were not directly assessed. No structured clinical sleep assessment, standardized sleep questionnaire, prospective sleep diary, actigraphy, or polysomnography was used. Napping, sleep phase, sleep efficiency, insomnia symptoms, circadian preference, and weekend compensatory sleep were also not systematically evaluated. Consequently, the study could not verify whether each participant's sleep duration was below his or her individual sleep requirement, and misclassification of sleep status in either group cannot be excluded (Matthews et al. 2018). Accordingly, HSS in this study should be interpreted as a self‐report‐based HSS classification rather than objectively verified sleep loss or a formal sleep‐disorder diagnosis, and the present EEG findings cannot be interpreted as biomarkers specific to objectively verified sleep loss.

The cross‐sectional design is another important limitation. The observed alpha‐band pattern may represent a predisposing trait, a state‐related correlate of clinically diagnosed GAD combined with self‐reported HSS, or both. Longitudinal studies will be needed to determine whether alpha‐band phase synchronization changes with symptom fluctuation, sleep recovery, or clinical intervention.

In addition, because the patient group consisted of individuals with both clinically diagnosed GAD and self‐reported HSS, the independent and interactive contributions of anxiety and sleep restriction could not be separated. A factorial design including GAD‐only, HSS‐only, combined GAD + HSS, and HC groups would be better suited to clarify whether the observed network pattern is more strongly associated with anxiety, sleep restriction, or their interaction.

Baseline differences also warrant caution. Age and years of education differed between groups at baseline. The nodal and graph‐theoretical findings remained after covariate adjustment, but residual confounding cannot be fully ruled out. Covariates were also not directly incorporated into the NBS model; the network‐level NBS result should therefore be interpreted as unadjusted with respect to these variables.

Finally, the analyses were conducted at the scalp level using a 30‐node EEG montage. Although CSD transformation and wPLI reduce the influence of volume conduction, they do not provide precise anatomical localization. The widespread component should therefore be interpreted as a scalp‐level functional connectivity pattern rather than direct evidence for specific cortical source networks. Source‐space connectivity analyses using high‐density EEG or MEG would help clarify the anatomical basis of this alpha‐band phase‐synchronization phenotype.

Overall, the present findings suggest that young adults with clinically diagnosed GAD and self‐reported HSS based on habitual weekday nocturnal sleep duration show a distributed alpha‐band phase‐synchronization pattern characterized by stronger weighted integration and clustering at rest. This pattern may reflect a less flexible resting‐state network configuration associated with hypervigilance, persistent internally oriented processing, and altered vigilance regulation. These interpretations remain hypothesis‐generating, but they extend the findings beyond a simple report of group differences and provide a testable network‐level framework for future studies of GAD combined with self‐reported HSS meeting a predefined HSS criterion.

## 5 Conclusion

In conclusion, young adults with clinically diagnosed GAD and self‐reported HSS based on habitual weekday nocturnal sleep duration showed altered resting‐state alpha‐band phase‐based connectivity compared with HCs. This group difference was observed in wPLI and was supported by convergent network‐level, nodal, and graph‐theoretical findings; the nodal and graph‐theoretical differences remained after adjustment for age and years of education. The results suggest a distributed alteration in alpha‐band phase coordination, characterized by stronger weighted integration and clustering at rest, rather than a nonspecific increase across connectivity measures. These findings should be interpreted as EEG connectivity differences associated with a clinically diagnosed GAD group meeting an HSS criterion, rather than evidence of objectively verified sleep loss‐specific mechanisms. Future studies using objective sleep assessment, longitudinal designs, GAD‐only and HSS‐only comparison groups, and source‐space connectivity analyses are needed to clarify the clinical and anatomical significance of this alpha‐band network pattern.

## Author Contributions

**Yajing Guo**: writing – review and editing, resources. **Mengyu Wang**: conceptualization, writing – review and editing, writing – original draft, methodology, project administration. **Ce Shi**: conceptualization, methodology, data curation, formal analysis, validation, visualization, writing – review and editing, writing – original draft, project administration. **Jing Wen**: writing – review and editing, resources. **Peidong Liu**: writing – review and editing, resources. **Yayong He**: writing – review and editing, resources. **Xinwang Chen**: methodology, supervision, resources, project administration, writing – review and editing. **Haiping Li**: writing – review and editing, resources. **Lihua Wu**: writing – review and editing, resources, supervision, validation, visualization, funding acquisition, writing – original draft, conceptualization. **Bingzhen Li**: writing – review and editing, resources. **Jing Gao**: resources, writing – review and editing. **Miaomiao Ji**: writing – review and editing, resources. **Run Zhang**: writing – review and editing, resources. **Wen Fu**: writing – review and editing, resources. **Cong Xue**: writing – review and editing, resources. Ce Shi and Mengyu Wang contributed equally to this work and share first authorship.

## Funding

This work was supported by the Henan Natural Science Foundation, Youth Science Fund (Category C), Grant no. 262300422240 and the 2026 Henan Science and Technology Development Program, Henan Science and Technology Research Project, Grant no. 262102310260.

## Ethics Statement

This study was approved by the Ethics Committee of the Third Affiliated Hospital of Henan University of Chinese Medicine (Approval No. 2023SH‐147). All procedures were conducted in accordance with the Declaration of Helsinki. Written informed consent was obtained from all participants prior to their enrollment in the study.

## Conflicts of Interest

The authors declare no conflicts of interest.

## Supporting information




**Supporting Material**: brb371685‐sup‐0001‐FigureS1.png

## Data Availability

The data that support the findings of this study are available from the corresponding author upon reasonable request.
